# Extending the reach of mousetracking in numerical cognition: a comment on Fischer and Hartmann (2014)

**DOI:** 10.3389/fpsyg.2014.01436

**Published:** 2014-12-10

**Authors:** Thomas J. Faulkenberry, Amandine E. Rey

**Affiliations:** ^1^Department of Psychology and Counseling, Tarleton State UniversityStephenville, TX, USA; ^2^Labaratoire Etudes des Mécanismes Cognitifs, Université Lumière Lyon 2Lyon, France

**Keywords:** numerical cognition, mousetracking, competition, dynamics, embodiment

In a recent article, Fischer and Hartmann (2014) present a brief methodological review of the use of computer mousetracking in analyzing the processes involved in numerical cognition. Most certainly this review is a welcome addition to the mathematical cognition literature, especially in light of recent studies that have used the technique to study numerical decision processes. After presenting a general overview of the computer mousetracking method, Fischer and Hartmann make several recommendations (e.g., reporting exact mouse settings, constraining wrist movement, etc.) that will surely help to facilitate comparison and interpretation across a variety of studies as we continue to advance our knowledge of the dynamics of numerical processing. The purpose of the present commentary is not to be critical; rather, we hope that this commentary will be seen as complementary to (as well as complimentary of) the recommendations of Fischer and Hartmann (2014). We feel that their review is timely and informative. However, we also feel that some of the issues raised by Fischer and Hartmann warrant further discussion.

At its core, computer mousetracking is used to construct a temporally rich set of data during decision-making that allows one to conduct a more fine-grained analysis than end-state performance measures alone (such as RT and/or error rates). Most of the recent studies that use this technique to study numerical processes specifically look for the dynamic signature of increased trajectory curvature in certain comparison conditions (e.g., Santens et al., 2011; Faulkenberry, 2014). This signature has been used as evidence for parallel consideration of response options. For example, Santens et al. (2011) measured trajectories in a numerical comparison task in which participants were asked to compare a presented number to the fixed standard 5. As the distance from the stimulus number to 5 decreased, trajectories became more differentially curved, revealing a dynamic interpretation of the numerical distance effect (Moyer and Landauer, 1967). Santens et al. interpreted their results to be in line with a competition-based model of numerical representations. Faulkenberry (2014) extended this result to a numerical odd/even task and showed (via distributional analyses of the response trajectories) that such differential curvatures result from a graded competition between parallel and partially-active representations, and not from averaging across widely different trajectory types. It is important to note that neither of these results could easily have been obtained via traditional cognitive processing measures.

It is on this note that we feel the review of Fischer and Hartmann (2014) unintentionally limits the utility of computer mousetracking to only providing evidence of continuous competition in numerical processing. On the contrary, several recent studies have used the technique to analyze the selective influence of various stimulus factors over the time course of a response. For example, Freeman and Ambady (2011) showed that trajectory deviations happen earlier for inconsistencies in pigmentation cues vs. shape cues in face recognition. Similarly, (Freeman et al., 2013) demonstrated earlier deviations for Chinese participants vs. American participants when processing faces with inconsistent contextual cues. While there are not yet any published studies in the domain of numerical cognition that look specifically at when trajectory deviations happen, Faulkenberry and Montgomery (2012) showed that in fraction processing, trajectory deviations which stem from components happen earlier than deviations which stem from holistic magnitude processing. The basic logic of these studies is that with an underlying mapping between response trajectories and perceptual/cognitive processes, any observed difference in the onset of motor deviations necessarily reflects a difference in the time course of the underlying perceptual and/or cognitive processes. As such, these types of manipulations hold promise for number researchers to tease apart the predictions from competing models of numerical processing.

There is one claim from which we hold a divergent opinion compared to Fischer and Hartmann (2014). Specifically, Fischer and Hartmann propose that competitive attraction from a distractor can be inferred *only* in the case where hand trajectories actually move the mouse completely onto the distractors side of the computer screen. Further, they propose that when trajectories remain completely on one side of the solution space, this “might simply reflect the earlier or later occurrence of the particpants' decisions, due to increased task difficulty” (Fischer and Hartmann, 2014, para. 7). This is in opposition to the continuous cognition framework (Spivey, 2007), which posits that any graded deflection of trajectories is due to attraction “toward” a competing response option (this is operationalized in terms of rising and falling activation values during a decision process). In fact, under this framework, increased task difficulty and increased competition are essentially synonymous. For example, (Gold and Shadlen, 2000) found when macaque monkeys were trained to indicate the perceived direction of a dot flow by making an eye movement toward that direction, the magnitude of eye movement deviations was modulated by the difficulty of perceiving the coherent dot flow direction. Similarly, (Santens et al., 2011) found that numerical distance modulated the curvature of hand trajectories in numerical comparison, even though those trajectories did not deviate into the competitors space. Both studies interpret these modulation effects as evidence of increased competition. Based on available evidence, we think that it is this increase of deviation/curvature in the presence of more difficult stimuli (or even a lack of deviation in a control condition) that serves as primary evidence for competition effects. Nevertheless, it is important for future research to investigate how the dynamics of hand trajectories reflect competition vs. indecision, and more theoretical work is needed to determine if/when such concepts can/should be dissociated.

Despite our objection to their claim, Fischer and Hartmann (2014) do raise a point worth further investigation. In those cases where hand trajectories do actually verge into the competitors space, what does this tell us? Further study may reveal additional explanations, but we offer one possibility. In a face recognition study, (Freeman et al., 2011) found that hand trajectories swerved toward the attribute that is stereotypically associated with the opposite sex when face cues partly overlapped with that sex (e.g., masculine women or feminine men). They interpreted this swerving as not only representing competing representations, but as a partial triggering of the associated stereotype. Hence, it could be the case that when hand trajectories do verge into the competing space, this says something about the nature of the competitive processes involved. Future research will need to investigate this issue more fully.

In conclusion, we appreciate and endorse the review of Fischer and Hartmann (2014). In spite of a few concerns that we have outlined above, we believe that their recommendations will be very influential, not only to number researchers, but to anyone hoping to use computer mousetracking to study real-time dynamics in cognition.

## Conflict of interest statement

The authors declare that the research was conducted in the absence of any commercial or financial relationships that could be construed as a potential conflict of interest.
